# Prediction of first-line immunotherapy response in patients with extensive-stage small cell lung cancer using a clinical-radiomics combined model

**DOI:** 10.3389/fimmu.2025.1688012

**Published:** 2025-12-12

**Authors:** Jing Fan, Xingyi Li, Jiamao Lin, Xiaopeng Song, Chenran Zhao, Fang Zhao, Zhenxiang Li

**Affiliations:** 1 Shandong First Medical University and Shandong Academy of Medical Sciences, Jinan, China; 2 Department of Radiation Oncology, Shandong Cancer Hospital and Institute, Shandong First Medical University and Shandong Academy of Medical Sciences, Jinan, China; 3 Department of Neurology, Heze Mudan District People’s Hospital, Heze, China; 4 Department of Internal Medicine, Shandong Cancer Hospital and Institute, Shandong First Medical University and Shandong Academy of Medical Sciences, Jinan, China; 5 Shandong Cancer Hospital and Institute, Shandong First Medical University and Shandong Academy of Medical Sciences, Jinan, China; 6 Department of Radiology, Qilu Hospital, Cheeloo College of Medicine, Shandong University, Jinan, Shandong, China

**Keywords:** ES-SCLC, CT image-based radiomics, TLS, immunotherapy, predictive model

## Abstract

**Objective:**

This study aimed to explore the value of clinical-radiomics features for predicting response to immunotherapy in extensive-stage small cell lung cancer (ES-SCLC).

**Methods:**

This retrospective study enrolled patients with ES-SCLC who received immunotherapy as first-line treatment from two centers. Patients were divided into a training and an external test cohort. Chest Computed Tomography (CT) images were obtained at baseline and after 2–3 cycles of immunotherapy. Each lesion was segmented based on intratumoral regions (ITR) in the plain scan (PS) and venous phase (VP) CT images. Radiomic features, including absolute and relative delta features were extracted. Four signatures were established by the least absolute shrinkage and selection operator (LASSO) after selecting relevant features. Multivariable logistic regression incorporating signature scores and clinical predictors was used to generate a nomogram. The performance of the nomogram was evaluated through area under the curves (AUC) analysis, calibration curves, and decision curve analysis (DCA). Tertiary lymphoid structures (TLS) and the tumor immune microenvironment (TIME) of tumors were investigated via multiplex immunohistochemistry (mIHC). Kaplan-Meier curves were constructed to illustrate Overall Survival (OS) in different patients groups.

**Results:**

The nomogram was built based on two radiomics signatures (ITR before treatment; relative delta radiomics) and two clinical factors (age; node). This model showed powerful predictive ability for both training and external test sets with AUCs of 0.919 and 0.839, respectively. Calibration curves and DCA showed a favorable predictive performance of the nomogram.

**Conclusion:**

The nomogram that included ITR, delta radiomic features, and clinical risk factors had the best performance in predicting prognosis for patients with ES-SCLC who received immunotherapy compared to models relying solely on radiomic features or clinical risk factors, and has the potential to assist clinicians in making personalized treatment decisions.

## Introduction

Platinum-based chemotherapy has been the standard treatment regimen for extensive stage small cell lung cancer (ES-SCLC) for decades (1). However, the majority of patients experience disease progression within 6 months and have limited treatment options after recurrence (2). Both the IMpower133 and CASPIAN clinical trials showed that chemoimmunotherapy as first-line treatment prolonged overall survival (OS) in patients with ES-SCLC (3, 4). Subsequently, randomized trials have been conducted in China for multiple Programmed Death-1(PD-1)/Programmed Death Ligand 1 (PD-L1) inhibitors including Serplulimab, Adebrelimab, Benmelstobart and Tislelizumab (5–8), collectively termed immune checkpoint inhibitors (ICIs). Despite OS improvements for patients with ES-SCLC treated with chemotherapy plus ICIs *vs* chemotherapy alone, efficacy has not met the standards set for patients with non-small cell lung cancer (NSCLC). The significant OS benefit observed in a subset of SCLC patients across multiple clinical trials underscores the existence of discriminative biomarkers for predicting immunotherapy response (9, 10).

PD-L1 expression and Tumor Mutation Burden (TMB) are associated with immunotherapy benefit in NSCLC and other solid malignancies; however, no predictive value has so far been demonstrated for chemoimmunotherapy in ES-SCLC (11). Although the CASPIAN trial showed better median OS in the durvalumab arm for patients with the inflamed/YAP1 subtype than other subtypes (12), the predictive power of transcriptomic SCLC subtyping remains unclear. Therefore, an accurate, accessible, and user-friendly predictive marker to assess the therapeutic efficacy of immunotherapy in ES-SCLC is urgently needed.

In the diagnosis and examination of lung cancer, tumors are routinely evaluated radiologically using CT, which captures the totality of the tumor and extracts a wide range of information. In recent years, the field of CT image-based radiomics has demonstrated considerable potential in lung cancer research, encompassing a broad spectrum of applications from early detection to prognostic assessment. It has been demonstrated that radiomics models can predict major pathological response in patients with NSCLC treated with immunotherapy (13). However, research on the value of CT image-based radiomics for predicting immunotherapy efficacy in ES-SCLC is less advanced. Moreover, novel advancements in Delta radiomics techniques have demonstrated their value for clinical applications in the diagnostic stratification and prognostic assessment of malignant tumors (14, 15).

Tertiary lymphoid structures (TLS) are transient, ectopic lymphoid aggregates capable of eliciting adaptive antitumor cellular and humoral immune responses. Within the tumor microenvironment, TLS are composed of T-cell zones and B-cell zones, predominantly localized in the stromal compartment (specifically in regions between tumor nests) mostly at the invasive margin of the tumor (16). First characterized in NSCLC as functional lymphoid formations linked to improved clinical outcomes, TLS have since been detected in various other malignancies, such as melanomas and sarcomas, where their presence correlates with enhanced immunotherapy responses (17, 18). Given their potential to improve survival in patients with cancer receiving ICIs, therapeutic strategies targeting TLS are now advancing toward clinical application. Thus, this multicenter, retrospective cohort study involved patients with ES-SCLC who received immunotherapy as their first-line treatment. We integrated radiomic and clinical features to develop a predictive model aimed at assessing the potential benefits of immunotherapy for different subgroups of patients with ES-SCLC. Thus, this multicenter, retrospective cohort study involved patients with ES-SCLC who received immunotherapy as their first-line treatment. We integrated radiomic and clinical features to develop a predictive model aimed at assessing the potential benefits of immunotherapy for different subgroups of patients with ES-SCLC.

## Methods

### Patients selection and clinical data collection

**Figure 1:**
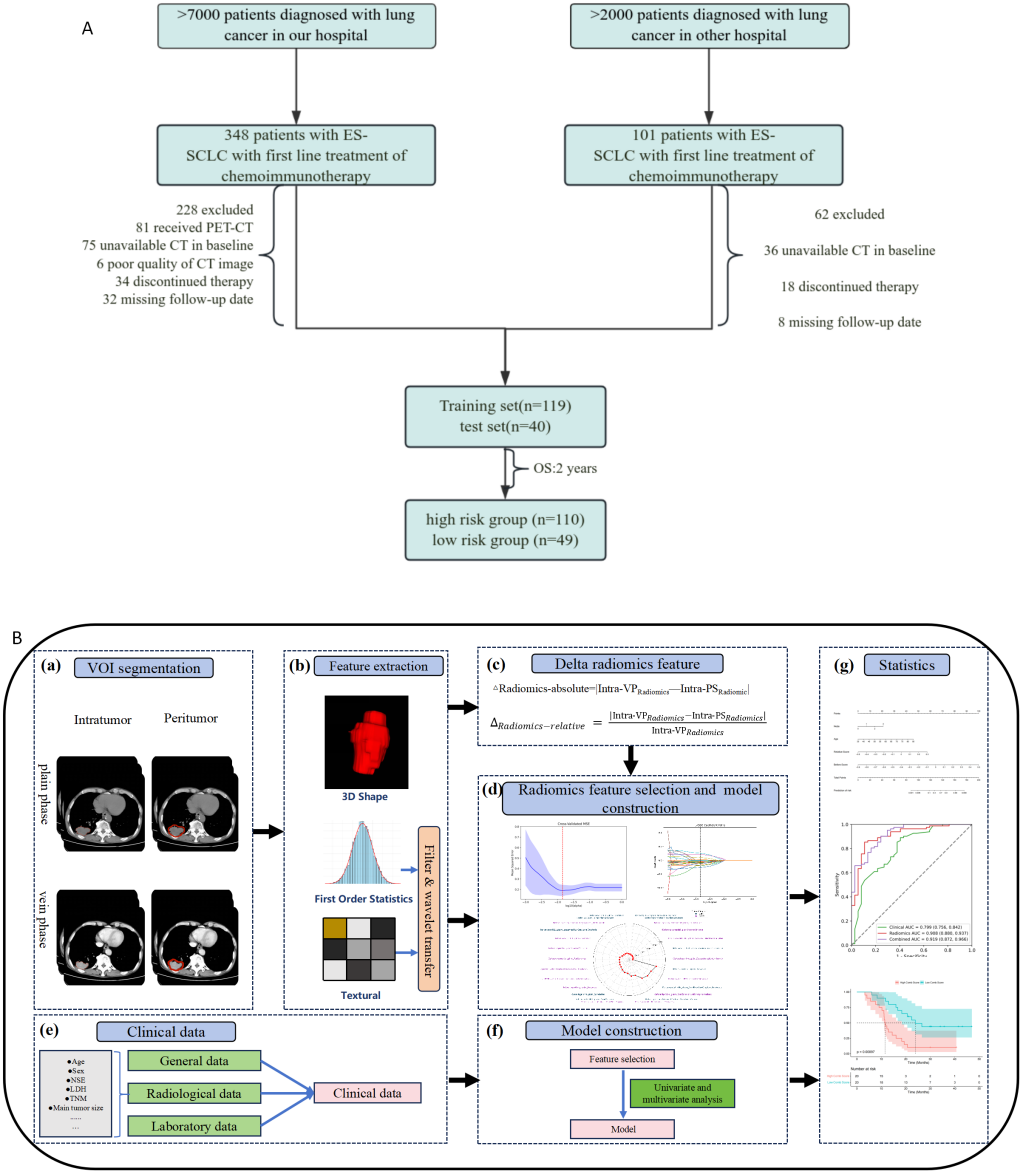
Study design. Flowchart of the patient selection process **(A)**; Workflow for radiomics feature extraction, model building, and analysis **(B)**. Registration CT images and VOI segmentation **(a)**; radiomics features including absolute and relative delta radiomic feature extraction **(b, c)**; radiomics features selection and model construction **(d)**; clinical date collection and model construction **(e, f)**; model building and test **(g)**.

In this retrospective study, we screened patients diagnosed with SCLC at our hospital between March 2020 and February 2024 as a training set. Patients were required to meet all of the following inclusion criteria: (1) Histologically confirmed SCLC classified as extensive stage according to the American Joint Committee on Cancer Eighth Edition, Tumor, Lymph node, and Metastasis staging system; (2) receipt of first-line standard chemoimmunotherapy (ICIs plus chemotherapy); (3) availability of chest CT scans at baseline and after 2–3 cycles of chemoimmunotherapy; (4) comprehensive clinical profiles such as sex, age at diagnosis, smoking history and administered therapies were fully documented. Exclusion criteria: (1) patients who received other therapeutic options before chemoimmunotherapy; (2) concurrent life-quality-limiting conditions (e.g., coexisting immune disorders, additional primary malignancies, etc.); (3) poor CT image quality, such as artefacts or quantum noise; (4) patients with missing follow-up data. Subjects meeting any single exclusion criterion were ineligible for study participation. A flowchart of patient inclusion and exclusion is depicted in **Figure 1A**.

The same inclusion and exclusion criteria were applied to patients from other centers as an external test cohort. Demographic information was collected as follows: age, smoking index, sex, Lactate Dehydrogenase (LDH) level at baseline (U/L), Neuron-Specific Enolase (NSE) level at baseline (ng/mL), Pro-Gastrin-Releasing Peptide (ProGRP) level at baseline (pg/mL), maximum diameter of tumor, TNM stage, baseline metastases, thoracic radiotherapy regimen, diagnosis date, date of diagnosis, and OS. OS was calculated from the date of ICI initiation to the date of death. The requirement for informed consent was waived due to the retrospective study design.

### Tumor segmentation

**Figure 1B** illustrates the procedural workflow implemented in our study. Segmentation of the volume of interest (VOI) in the plain scan (PS) and venous phase (VP) scan CT images was carried out by two proficient radiologists (Z.F. and L.H.Y., with 11 and 7 years of experience in CT image interpretation, respectively). Both radiologists were blinded to all clinical and pathological data to minimize bias. In instances of disagreement, a third radiologist (P.G.D, with 15 years of experience in thoracic imaging) intervened to facilitate discussion and resolution.

The intratumoral region, as depicted in **Figure 1B**, was defined as the primary SCLC and meticulously contoured on axial contrast-enhanced computed tomography (CECT) layer by layer using ITK-SNAP 3.8.0 software (www.itksnap.org).

### Radiomics feature extraction

Radiomic features were extracted from the intratumoral regions in the PS and VP CT images using PyRadiomics (version 3.1.0, https://pyradiomics.readthedocs.io/), a Python-based library. A normalization procedure using PyRadiomics was applied to the CT images before feature extraction to enhance the repeatability of radiomic analyses.

These features were categorized into 3D shape features, first-order statistics, and texture features. Comprehensive details regarding the feature categories are available in **Supplementary Note S1**. All extracted radiomic features were compliant with the Initiative for Image Biomarker Standardization criteria (IBIS, https://theibsi.github.io) (19). Furthermore, the values of these radiomics features were standardized using the *z*-score method.

The intratumoral radiomic features were systematically extracted from tumor regions delineated on both PS and VP CT images, designated as intra-PS and intra-VP radiomic features, respectively. We operationalized Delta radiomics through dual computational approaches:

(i) The absolute variation in each radiomic feature quantified by arithmetic subtraction (Intra-VP – Intra-PS) (20):

(1) $$\Delta_{Radiomics-absolute}=|\text{Intra‐}{\text{VP}}_{Radiomics}-\text{Intra‐}{\text{PS}}_{Radiomics}|$$

(ii) The relative variation of each radiomic feature was calculated as follows (21):

(2) $$\Delta_{Radiomicsrelative-|}\frac{\left|\text{Intra‐}{\text{VP}}_{Radiomics}-\text{Intra‐}{\text{PS}}_{Radiomics}\right|}{\text{Intra‐}{\text{VP}}_{Radiomics}}$$

### Radiomics feature selection

Techniques for selecting dimensions were utilized to reduce redundancy and decrease dimensionality that can result from overfitting and bias during the construction of radiomic features. A four-step methodology was employed to lower the dimensionality of high-dimensional data while filtering the feature input for the machine learning model. First, the reproducibility of VOI delineation was evaluated through interclass correlation coefficient (ICC) analysis, focusing on both intra-observer and inter-observer variability. Thirty randomly selected SCLC patients underwent repeat VOI segmentation after 1 month by two radiologists (Z.F. and L.H.Y.). Only radiomics features with an ICC greater than 0.8 were included in subsequent analyses. Following this, a variance threshold of 0.8 was applied to further refine feature selection. The one-way analysis of variance (ANOVA)-based SelectKBest algorithm was employed to select features with P values less than 0.05 for subsequent analysis. Finally, the LASSO was used for the optimal construction of a radiomics feature subset in the training set. The model underwent 10-fold cross-validation on the training cohort to determine the optimal regularization strength (a), with selection criteria based on minimum mean squared error.

### Model development and validation

The multivariate regression coefficients (a) derived from LASSO regularization were employed as feature weights. Each patient’s radiomic signature (RadScore) was computed through linear combination of z-score normalized features. The final radiomic RadScore for each subject was computed as:


$$\text{RadScore}=\sum_{i=1}^{n}\beta_{i}X_{i}$$

where $\beta_{i}$ represents the LASSO-derived coefficient for the i-th selected feature, and $X_{i}$ denotes the z-score normalized feature value.

Clinical factors and radiomics signatures were integrated into univariate and multivariate logistic regression analyses. To identify independent risk factors, multivariate logistic regression with backward stepwise selection, guided by the minimal Akaike information criterion, was utilized. A nomogram was constructed using the chosen variables. Additionally, for comparison purposes, a clinical model utilizing only the selected clinical characteristics and a radiomics model based on the identified radiomics signatures were developed within the training set. The predictive performance of the models was evaluated by receiver operating characteristic (ROC) curve analysis, and AUC, sensitivity, specificity, and accuracy were calculated respectively. DCA was employed to evaluate the net benefit of the models in a clinical context.

### Statistical analysis

The R software (version 4.2.1; https://www.rproject.org/) was utilized to conduct statistical analyses. Continuous variables with a normal distribution are presented as mean ± standard deviation (SD); otherwise, they are expressed as median with interquartile range (IQR). Categorical variables are described using frequencies and percentages. The Wilcoxon rank-sum test, Kruskal-Wallis test, χ² test, or Fisher’s exact test was applied to compare variables between the training and external test cohorts. ROC curve and DCA along with the AUC were created to evaluate the predictive capability of the models. Additionally, calibration curves were developed through 1,000 iterations of bootstrap resampling. All statistical tests were conducted two-sided, with P values less than 0.05 considered statistically significant. Kaplan-Meier survival curves were produced and compared using the log-rank test.

### Multiplex immunohistochemistry and multichannel imaging

Tumor biosamples at baseline were obtained for mIHC to assess the distribution and composition of TLS within tumors, and four molecular subtypes of SCLC were defined by their distinct transcriptional regulators. Deparaffinization of tissue sections was done through xylenes and rehydration through decreasing graded alcohol. AR6 buffer (Akoya Biosciences) was used for antigen retrieval in a microwave oven. Endogenous peroxidase was inactivated by incubation in 3% H2O2 for 10 min. Multiplex immunohistochemistry was performed by several rounds of staining, each including a protein block with 1% BSA followed by primary antibody and corresponding secondary horseradish peroxidase-conjugated antibody against mouse or rabbit immunoglobulins (Akoya Biosciences). The slides were then incubated in different Opal fluorophore (1:100) diluted in 1X Plus Amplification Diluent (Akoya Biosciences). After tyramide signal amplification and covalent linkage of the individual Opal fluorophores (Akoya Biosciences) to the relevant epitope or epitopes, the primary and secondary antibodies were removed via antigen retrieval as previously mentioned and the next cycle of immunostaining was initiated. Primary antibodies are presented in **Supplementary Table S1**. All slides were counterstained with spectral DAPI (Akoya Biosciences) and mounted with Anti-fade fluorescence mounting medium (ab104135, Abcam). Multichannel imaging was performed on a PANNORAMIC SCAN II Imaging System (3Dhistech, Hungary) and slides were imaged at ×200 magnification.

## Results

### Patients characteristics and treatment outcomes

A total of 449 patients with ES-SCLC were initially screened, and our longitudinal follow-up study of some patients demonstrated a median overall survival (mOS) of 18.8 months. Survival curves demonstrated a steep decline in survival rates during the first 2 years, followed by a plateau phase, indicating that patients who survive beyond 2 years may attain substantially longer OS than those who do not (**Supplementary Figure S1**). Based on observed survival curve plateaus and clinical relevance, we set 2 years as the optimal prognostic cutoff value for subsequent analyses. Of these 449 patients, 198 were excluded due to inaccessible CT images or poor image quality, 40 were excluded for missing follow-up data, and 52 were excluded due to discontinuation of treatment. Ultimately, 159 patients were enrolled and allocated into the training set (n=119) and the test set (n=40). The median age at baseline was 60.73 years (range: 38–82) in the training set and 61.15 years (range: 43–74) in the test set. Both cohorts were predominantly male, comprising 81% (n=96) and 80% (n=32) of the training and test sets, respectively. Additionally, a majority of patients in both groups had a history of smoking (n=67 in the training set [56%] and n=21 in the test set [52%]). All participants had stage IV cancer at enrollment. At baseline, metastases were observed in the following sites: Training set: (26%, n=31), liver (35%, n=42), bone (29%, n=34). Test set: brain (28%, n=11), liver (45%, n=18), bone (33%, n=13). Based on OS, patients were stratified into a high-risk group (OS < 2 years; n=110, with 82 in the training set and 28 in the test set) and a low-risk group (OS ≥ 2 years; n=49, with 37 in the training set and 12 in the test set). The high-risk group suggested a potentially poorer response to immunotherapy, whereas the low-risk group indicated a more favorable prognosis. A positive event was defined as the induction of a significant immune response in this study. Detailed baseline characteristics are presented in **Table 1**.

**Table 1:** The clinical and imaging characteristics of patients in the training and test cohorts.

Characteristics	Training set (n = 119)	Test set (n = 40)	P value
Age, Mean ± SD	60.73 ± 8.93	61.15 ± 7.03	0.762
sex, n (%)			1
Men	96 (80.7)	32 (80.0)	
Female	23 (19.3)	8 (20.0)	
Smoking, n(%)			0.814
Never	52 (43.7)	19 (47.5)	
Smoker	67 (56.3)	21 (52.5)	
LDH, Median (Q1,Q3)	241 (172.5, 300)	270 (202.8, 362.3)	0.087
NSE, Median (Q1,Q3)	62.7 (31.8, 97.6)	60.55 (28.6, 91.6)	0.558
ProGRP, Median (Q1,Q3)	921 (594.6, 2232.5)	921 (276.5, 2251.5)	0.656
ICIs			0.495
Durvalumab	29 (24.4)	12 (30.0)	
Atezolizumab	20 (16.8)	7 (17.5)	
Serplulimab	11 (9.2)	8 (20.0)	
Adebrelimab	21 (17.6)	6 (15.0)	
Tislelizumab	12 (10.1)	1 (2.50)	
Benmelstobart	16 (13.4)	3 (7.5)	
Investigational Product (PD-L1 inhibitor)	10 (8.4)	3 (7.5)	

LDH, lactate dehydrogenase; NSE, neuron-specific enolase; ProGRP, pro-gastrin-releasing peptide; ICIs, immune checkpoint inhibitors; PD-L1, programmed death-ligand 1.

During the treatment course, 63 patients (n=50 in the training set and n=13 in the test set) received thoracic intensity-modulated radiation therapy (IMRT), the target volume predominantly covering primary pulmonary lesions combined with the involved regional lymph nodes. The prescribed radiation dose for all individuals was above 30 Gy. Thoracic consolidation radiotherapy (TRT) showed a trend toward an OS benefit compared to systemic therapy alone, although this did not reach statistical significance (P = 0.073; **Supplementary Figure S2**). These findings are consistent with previously reported randomized controlled trials (22), although the optimal timing of thoracic radiotherapy, as well as the appropriate radiation dose and fractionation regimen, require further investigation, and their safety profiles need to be thoroughly evaluated. Among patients with baseline brain metastases, 33 received cranial radiotherapy, primarily intensity-modulated radiation therapy (IMRT). The radiation fields mostly combined whole-brain radiotherapy (WBRT) with simultaneous integrated boosts (SIB) to metastatic foci, with a definitive radiation dose ≥30 Gy. In the non-brain-metastatic cohort, 6 patients underwent prophylactic cranial irradiation (PCI) according to institutional protocol (typically 30 Gy in 10 fractions). The cumulative incidence of subsequent brain metastasis was 17% (1/6), with the sole case occurring in a high-risk patients.

Treatment response after 2–3 cycles of chemoimmunotherapy demonstrated: partial response (PR): 61% (n=97; high-risk: n=67, low-risk: n=30); stable disease (SD): 38% (n=60; high-risk: n=43, low-risk: n=17); progressive disease (PD): 1% (n=2, both high-risk). During follow-up, disease progression occurred in 128 patients, with the following patterns: isolated primary site progression: 45% (n=58); distant metastasis alone: 45% (n=58); combined primary and distant progression: 2% (n=2); biochemical progression (tumor marker elevation only): 6% (n=8); unknown progression pattern: 2% (n=2).

### Radiomics feature extraction

**Figure 2:**
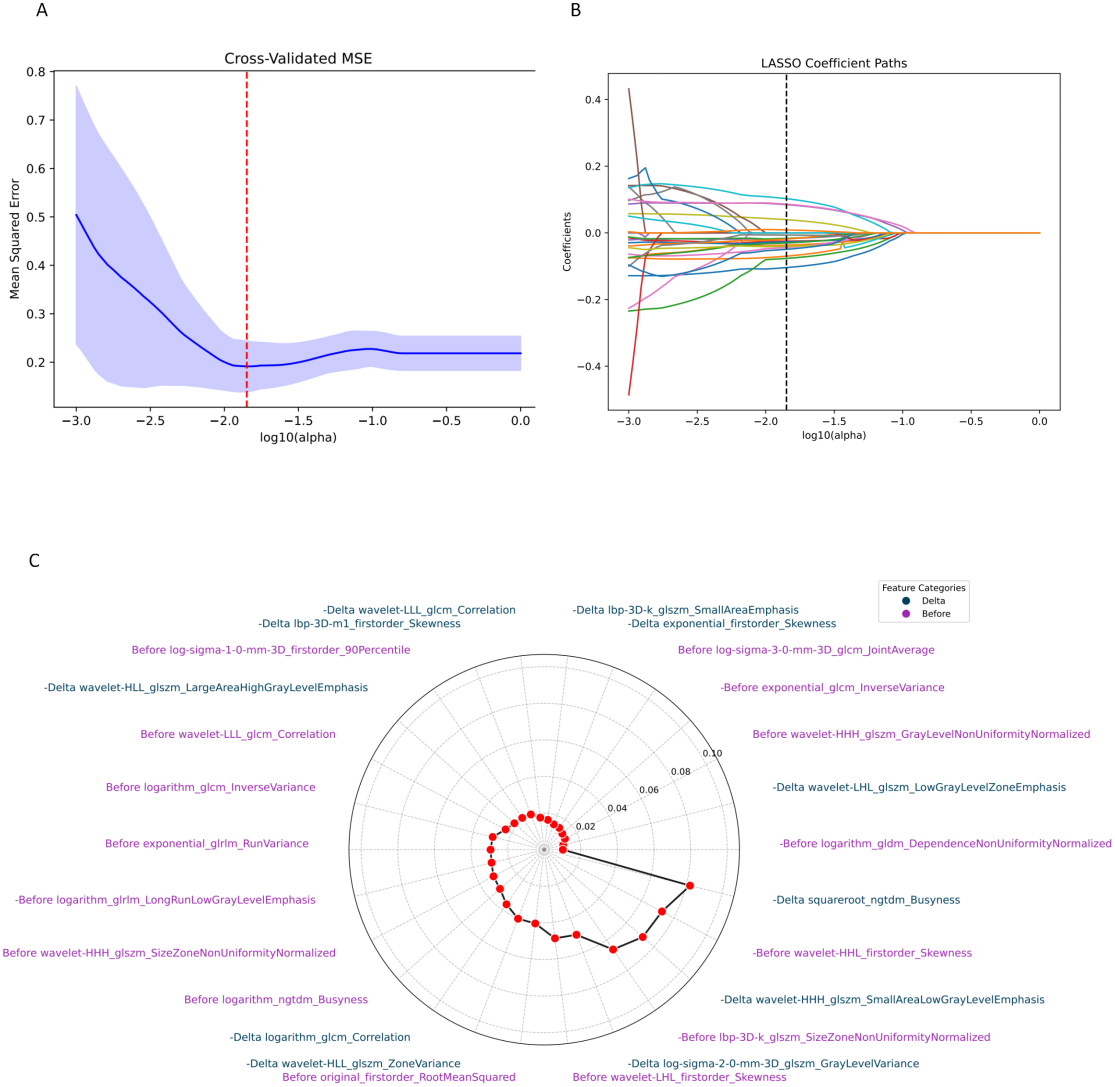
Radiomics feature screening. A plot of MSE screened with relative Delta feature least absolute shrinkage and selection operator **(A)**; corresponding to the non-zero feature **(B)**; visualisation of Delta and before-treatment features **(C)**.

A total of 1,688 radiomics features were meticulously extracted from the tumor interior regions of PS and VP CT images. Based on **Equations 1** and **2**, this ultimately yielded 1,674 absolute Delta features and 1,674 relative Delta features. Ultimately, a total of 6724 (1688×2 + 1674×2) radiomics features (ANOVA) was used to screen features with a P-value less than 0.05 for subsequent analysis. Then, LASSO was used to further select features, and 15, 6, 9 and 11 features were selected as the most valuable features and to construct signatures for ITR before- and after treatment, absolute, and relative delta radiomics, respectively. The detailed features and their respective coefficients are shown in **Figure 2**.

### Performance of radiomics models alone

**Figure 3:**
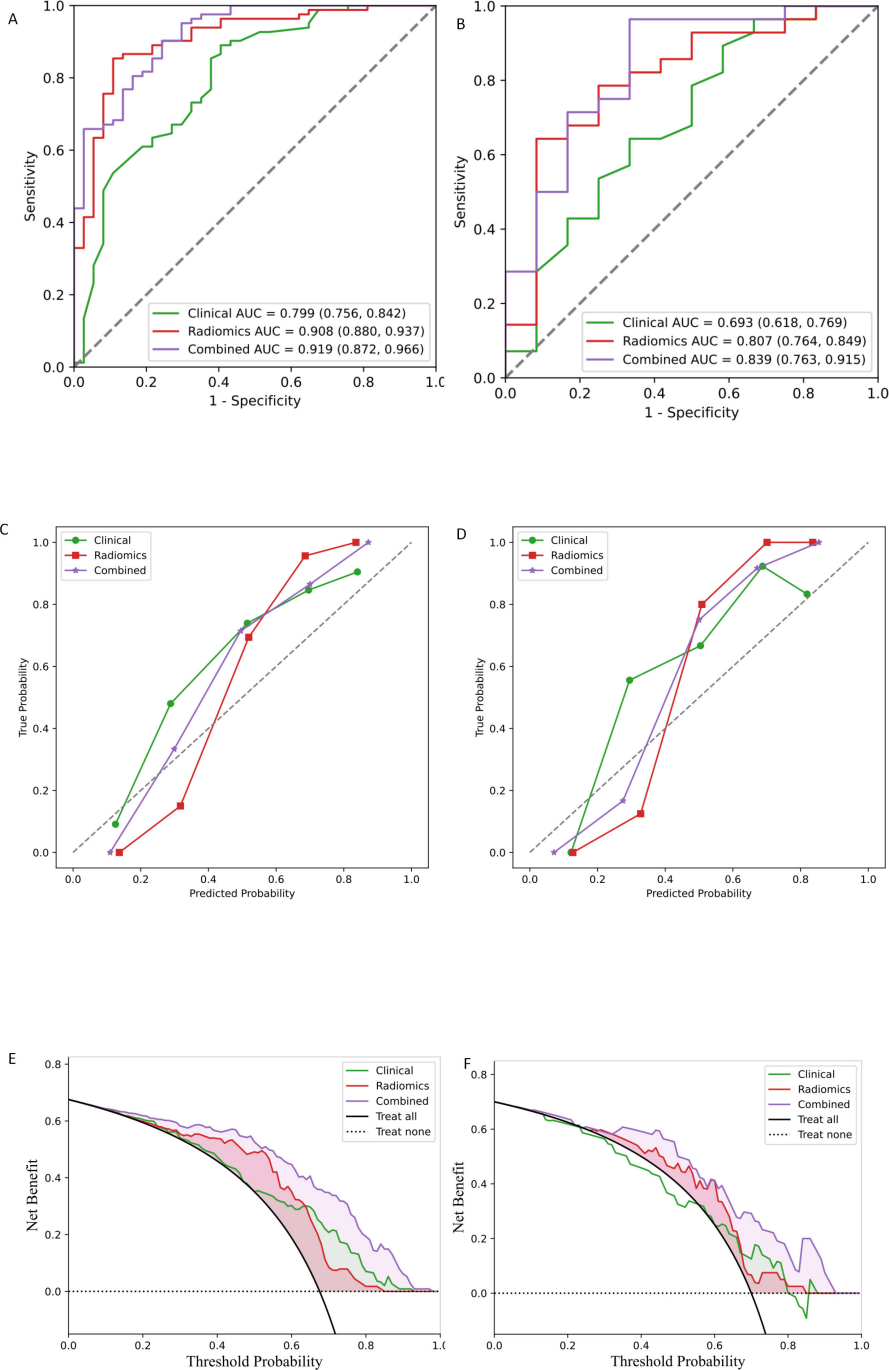
Assessment of models for the prediction of immunotherapy efficacy. Areas under the receiver operating characteristic curve (AUCs) for the training set **(A)** and test set **(B)**. Calibration curve for the training set **(C)** and test set **(D)**. Decision Curve Analysis (DCA)for the training set **(E)** and test set **(F)**. Red lines represent the radiomics model, green lines represent the clinical model, and purple curves represent the clinical combined radiomics model.

Univariate analysis of the training set identified that before-treatment radiomics scores, after-treatment radiomics scores, absolute delta radiomics scores and relative delta radiomics scores were associated with immune response (**Table 2**). The four radiomics models (before-treatment, post-treatment, absolute delta, and relative delta) incorporated features extracted from both PS and VP images, and model performance was evaluated using ROC curve analysis. Before-treatment model: training set AUC = 0.882, test set AUC = 0.476. After-treatment model: training set AUC = 0.735, external test set AUC = 0.565. Absolute delta model: training set AUC = 0.799, test set AUC = 0.598. Relative delta model: training set AUC = 0.791, test set AUC = 0.738. Beyond the AUC, radiomics model’s sensitivity, specificity, and accuracy are further detailed in **Table 3**. Multivariable analysis revealed radiomics predictors: before-treatment radiomics scores (OR = 3.244, 95% CI:1.838-6.648; P<0.001), relative delta radiomics scores (OR = 2.866, 95% CI:1.510-6.403; P = 0.004) (**Table 2**). The final radiomics model was constructed using features selected through multivariable analysis and demonstrated superior performance compared with the above-mentioned univariate radiomics models: training set: AUC = 0.908 (95% CI: 0.880-0.937), test set: AUC = 0.807 (95% CI: 0.764-0.849) (**Figures 3A, B**). The final radiomics model’s sensitivity, specificity, and accuracy are detailed in **Table 4**.

**Table 2:** Logistic regression analysis of variables for association with immune efficacy in patients in the training set.

Characteristics	Univariable analysis		Multivariable analysis	
	OR	P Value	OR	P Value
Before-Score	1.952	<0.001	3.244	<0.001
After-Score	1.615	<0.001	1.064	0.871
Absolute-Score	1.736	<0.001	1.249	0.352
Relative-Score	2.246	<0.001	2.866	0.004
Age	1.064	0.004	1.168	0.002
NSE at baseline	1.005	0.036	1.002	0.658
LDH at baseline	1.003	0.022	0.998	0.370
Tumor stage	1.339	0.045	0.961	0.894
Lymph node stage	1.956	0.014	2.917	0.046
Metastases stage	1.590	0.044	0.802	0.780
Gender	1.553	0.291	NA	NA
BMI	1.006	0.895	NA	NA
KPS	9.470	0.058	NA	NA
Smoking index	1.000	0.576	NA	NA
Maximum diameter of primary focus	1.000	0.994	NA	NA

BMI, Body mass index.

**Table 3:** Diagnostic performance of radiomics models.

Model	Features	Training set				Test set			
		AUC	Sensitivity	Specificity	Accuracy	AUC	Sensitivity	Specificity	Accuracy
Before-treatment	15	0.882	0.780	0.784	0.782	0.476	0.750	0.333	0.625
After-treatment	6	0.735	0.683	0.703	0.689	0.565	0.643	0.417	0.575
Absolute delta	9	0.800	0.695	0.703	0.697	0.598	0.679	0.583	0.650
Relative delta	11	0.791	0.768	0.595	0.714	0.738	0.643	0.667	0.650

AUC, the area under the curve.

**Table 4:** Comparative diagnostic performance of final radiomics, clinical, and combined models.

Model	Features	Training set				Test set			
		AUC	Sensitivity	Specificity	Accuracy	AUC	Sensitivity	Specificity	Accuracy
Final radiomics	2	0.908	0.890	0.757	0.849	0.807	0.857	0.583	0.775
Clinical	2	0.799	0.732	0.649	0.706	0.693	0.679	0.500	0.625
Combined	4	0.919	0.841	0.784	0.824	0.839	0.857	0.667	0.800

AUC, the area under the curve.

### Establishment of the combined prediction model

Univariate analysis of the training set identified that age, baseline LDH level, baseline NSE level, Tumor stage (T-stage), Lymph node stage (N-stage), and Metastasis stages (M-stage) were clinical variables associated with immune response. Multivariate analysis revealed age (OR = 1.168, 95% CI:1.068-1.305; P value=0.002) and Lymph node stage (OR = 2.917, 95% CI:1.039-9.025; P value=0.046) as additional predictors of immune response (**Table 2**). Clinical prediction models were constructed using multivariate logistic regression analyses along with age and N-stage as core clinical predictors; the AUC for the clinical model was 0.799 in the training set and 0.693 in the test set (**Figures 3A, B**). The clinical model demonstrated sensitivity, specificity, and accuracy of 0.732, 0.649, and 0.756 in the training set, and 0.679, 0.500, and 0.625 in the test set, respectively (**Table 4**). Compared with radiomics models, which show significant potential as independent predictors, clinical prediction models have limited accuracy, prompting the development of a comprehensive model that combines clinical and radiomics features. The combined model was constructed by integrating two clinical features and two radiomics features selected through multivariate logistic regression analysis. Compared with the simple radiomics model and clinical model, the AUC values of the combined prediction model were improved: training set AUC = 0.919 (Δ+0.120 *vs* clinical, +0.011 *vs* radiomics), test set AUC = 0.839 (Δ+0.146 *vs* clinical, +0.032 *vs* radiomics) (**Figures 3A, B**). In the test cohort, the combined prediction model demonstrated superior sensitivity, specificity, and accuracy compared to both the standalone radiomics model and clinical prediction model (**Table 4**). The combined model showed significant improvement in discriminative ability compared to individual models, as evidenced by increased integrated discrimination improvement values in both training and test cohorts.

**Figure 4:**
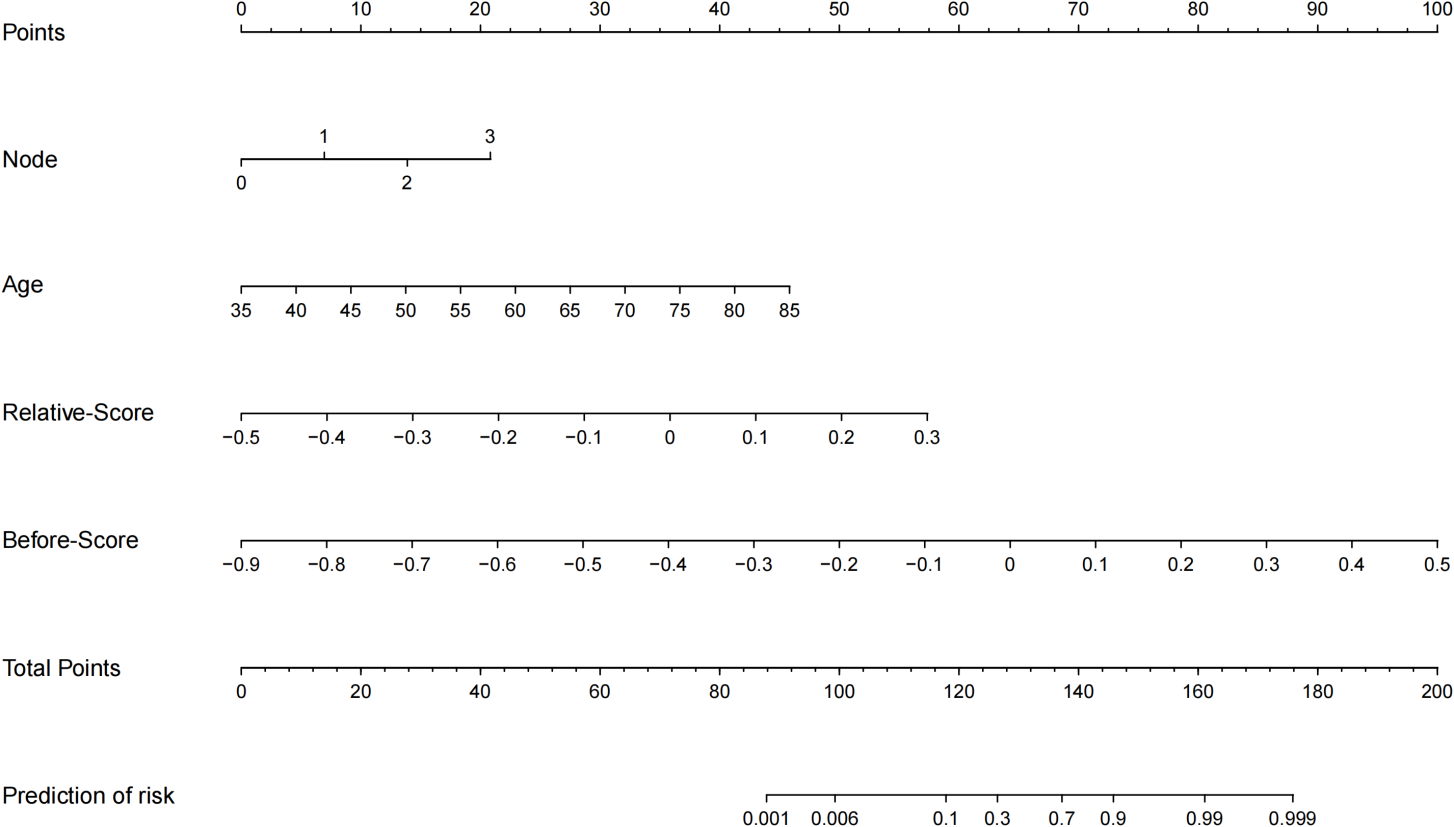
Nomogram of the combined model for predicting immunotherapy efficacy in patients with ES-SCLC.

**Figure 5:**
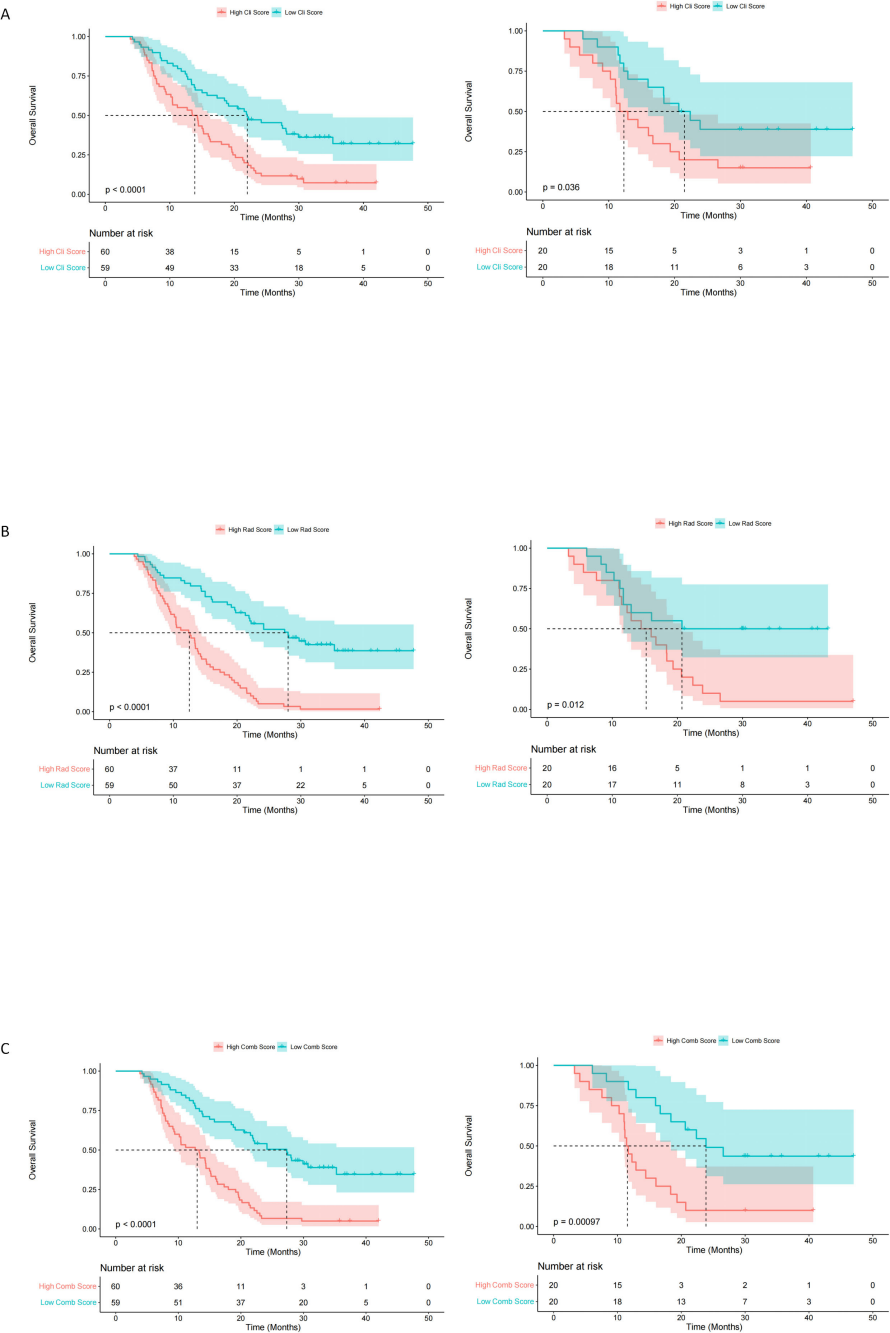
Kaplan-Meier curves representing overall survival in the outcome cohort. Clinical-score **(A)**; Radiomics-score **(B)**; Combined-score **(C)**, the left side is the training set and the right side is the test set. High model score is represented by the red line and low model score is represented by the blue line.The high and low groups were divided into equal numbers based on the median of the model-driven scores. P values were calculated using a two-sided log-rank test. The Clinical-score, Rad score and hybrid score were derived from the clinical model, radiomics model and combined model, respectively.

Calibration analysis revealed suboptimal agreement between the probability of immune efficacy predicted by the model and the observed probability (**Figures 3C, D**). DCA demonstrated that the combined model offered superior clinical utility, yielding greater net benefits across the spectrum of reasonable threshold probabilities when compared to the radiomics model alone (**Figures 3E, F**). The final nomogram effectively visualized variable contributions (**Figure 4**). The clinical-score, radiomics-score, and combined score were associated with overall survival; the K­M curves for OS demonstrated a statistically significant separation beginning at an early stage and persisting until the final observation date (**Figure 5**).

### TLS and TIME: their correlation with survival and predictive modeling

We performed correlation analyses between TIME and radiomic features, aiming to elucidate the molecular mechanisms underlying the radiomics-based predictive model. We collected baseline biological specimens from 33 enrolled patients for mIHC; detailed detection indicators are provided in **Supplementary Table S1**.

We did not identify any significant associations between TLS and either OS using our combined model (**Supplementary Figure S3**). We next focused on characterizing the tumor microenvironment. Using median proportions of immune cells across different spatial structures as cutoff values, the cohort was stratified into high- and low-expression subgroups. Survival analysis revealed that infiltration of CD23^+^ immune cells in tumor regions was significantly associated with longer OS (P value=0.018, **Supplementary Figure S4A**). However, Fisher’s exact test demonstrated no significant correlation between CD23^+^ immune cell infiltration and the high-/low-risk classification of our combined predictive model (**Supplementary Figure S4B**). Other immune cells in different spatial structures showed trends toward survival association (**Supplementary Figure S5**) but failed to reach statistical significance. Next, patients were classified into high- and low-risk groups based on the combined score to compare differences in immune microenvironment composition and expression. While most immune cell subsets showed no significant differences between risk groups, stromal CD8^+^ T cell infiltration exhibited an significant trend (P = 0.040, **Supplementary Table S2**). Subsequently, patients were divided into high/low CD8^+^ T cell expression groups based on median stromal CD8^+^ T cell infiltration levels. Fisher’s exact test revealed no statistically significant association between stromal CD8^+^ T cell density and stratified combined risk score (P value = 0.118) (**Supplementary Figure S6**).

## Discussion

Our findings demonstrate that radiomic features serve as robust independent predictors of immunotherapy response. Notably, the integration of clinical parameters with radiomic signatures significantly enhanced our model’s predictive performance compared to separate clinical or radiomics models., suggesting this multimodal approach may offer superior clinical utility for treatment stratification in patients with ES-SCLC. Currently, first-line treatment guidelines for ES-SCLC recommend chemoimmunotherapy based on the landmark IMpower133 and CASPIAN trials. However, these studies demonstrated only modest progression-free survival (PFS) benefits and more substantial OS advantages, suggesting immunotherapy may exert a ‘long tail effect’ by sustaining durable responses in a subset of patients. This dissociation between PFS and OS outcomes underscores the importance of prioritizing OS evaluation when assessing first-line treatment efficacy. Given these considerations, we selected OS as the grouping criteria, as it more accurately reflects the true clinical benefit of immunotherapy in ES-SCLC and better captures its potential long-term survival advantages. Based on long-term follow-up data from pivotal clinical trials (9, 10, 23), the survival curve of the chemoimmunotherapy group demonstrates a plateau phase after 2 years, with a markedly attenuated decline in survival rates. This observed pattern shows strong concordance with the survival dynamics identified in our study, validating the scientific rationale for selecting 2-year survival as the cutoff value for efficacy assessment.

Although numerous studies have focused on biomarkers of immunotherapy in SCLC, there is a lack of clinically applicable biomarkers in SCLC. A study by Shames et al. looked at comprehensive characterization of the significant immune heterogeneity in SCLC. In their analysis of the IMpower133 trial cohort, patients were stratified into neuroendocrine and non-neuroendocrine phenotypes based on established gene expression pathways and prior subtyping classifications. The study revealed a striking differential response to immunotherapy: neuroendocrine tumors demonstrating low tumor-associated macrophage (TAM) infiltration but high T-effector signals showed significantly prolonged OS when treated with atezolizumab plus etoposide-platinum (EP) chemotherapy compared to EP alone. This therapeutic benefit contrasted sharply with non-neuroendocrine tumors, which exhibited both high TAM infiltration and elevated T-effector signals (24). These findings underscore the complexity of the TIME in SCLC, providing a mechanistic explanation for the limited efficacy of current immunopredictive markers. Additionally, previous efforts to identify predictive biomarkers for immunotherapy typically required invasive procedures to obtain tissue samples, with sample purity, stability, and quantity being critical to ensuring the reliability of results. These cumulative limitations significantly constrain the clinical utility of current biomarker approaches. Our integrated prognostic model, which combines chest CT imaging and clinical characteristics, offers a cost-effective and non-invasive solution to accurately identify patients with ES-SCLC who are likely to respond well to immunotherapy. Unlike biopsy samples, which only provide localized information, CT imaging captures comprehensive tumor features, including peritumoral vascular responses and immune activity. Delta radiomics emphasizes the measurement of temporal changes in quantitative imaging biomarkers. This method enables the evaluation of longitudinal shifts in tumor radiomic characteristics using dynamic contrast-enhanced imaging. It indicates the relative net modification in radiomic features as a consequence of dynamic variations in angiogenesis, thus illustrating changes in tumor heterogeneity and aggression. However, absolute delta and post-treatment radiomics failed to demonstrate satisfactory predictive performance in our study. This limitation may be attributed to the inherent chemosensitive characteristics of SCLC. Following 2–3 courses of chemoimmunotherapy, most patients had rapid tumor regression, making lesions difficult to measure and consequently challenging to extract stable radiomic features.

Existing ICIs primarily exert their potent anti-tumor effects by blocking the PD-1/PD-L1 pathway, thereby reactivating functionally impaired or exhausted cytotoxic T lymphocytes (CTLs) (25, 26). This mechanism enables robust restoration of CTL effector functions, ultimately leading to sustained control of tumor progression. However, only a small portion of patients with ES-SCLC respond to ICI treatment. This is because SCLC is generally characterized by an immunologically ‘cold’ TIME (27). Importantly, the SCLC TIME is heterogeneous and exhibits dynamic interplay between immune and tumor cells, featuring spatially and temporally variable interactions that can paradoxically both suppress and promote tumor progression (28).

In this study, after validating the combined model’s predictive capacity for identifying potential immunotherapy responders, we further investigated potential correlations between the model predictions and both the TIME and TLS. However, no statistically significant associations were observed, which may be attributed to the limited biological sample size and the high heterogeneity of SCLC.

A previous study that used spatial multi-omics integration to map the single-cell landscape of SCLC found that multiple positive tumor cells (MPTCs), subtype interconversion, and intercellular interactions exhibit distinct patterns among different cellular subtypes (29). The high intratumoral diversity and spatial heterogeneity in SCLC significantly complicate the exploration of predictive biomarkers for immunotherapy efficacy. Nevertheless, negative correlation results demonstrate the potential advantages of radiomics in terms of stability and reproducibility TIME assessment, providing important methodological insights for future research.

Our study has several limitations that should be acknowledged. First, as a retrospective analysis, it is susceptible to incomplete data and recall bias. Second, although we endeavored to include as many patients as possible during model construction, the final cohort remained relatively small due to stringent inclusion criteria, and the number of pathological specimens obtained was even more limited. The small sample size and restricted biological samples may introduce potential statistical bias, an issue further compounded by heterogeneity in treatment regimens (i.e., variations in drug administration among patients). Third, PFS and post-treatment laboratory parameters were not analyzed. Moreover, to balance model simplicity and clinical utility, pathological indicators such as Ki67 proliferation index were not included, and mechanistic exploration of immune pathways was lacking. In future research, we plan to expand the patient cohort, collect more biological samples, and integrate multi-dimensional data to enhance the comprehensiveness of the model. We believe these improvements will contribute to building more robust and clinically applicable research models in the near future.

## Data Availability

The original contributions presented in the study are included in the article/**Supplementary Material**. Further inquiries can be directed to the corresponding authors.
