# Supplementary material for: Prediction of first-line immunotherapy response in patients with extensive-stage small cell lung cancer using a clinical-radiomics combined model
Source: Front Immunol. 2025 Dec 12;16:1688012. doi: 10.3389/fimmu.2025.1688012 (PMC12741101; doi:10.3389/fimmu.2025.1688012)
Supplement: Supplementary file 1 [file Table1.docx]

**Supplementary Note 1** Radiomics feature extraction details.

1. Texture Feature Classes

Gray-level size zone matrix (GLSZM)

Gray-level run-length matrix (GLRLM)

Gray-level co-occurrence matrix (GLCM)

Gray-level dependence matrix (GLDM)

Neighborhood gray-tone difference matrix (NGTDM)

2. Multiscale Filter Bank

Shape features were computed from original images, while other features were generated using:

Wavelet transform (8-directional decomposition)

Square transform

Square root operation

Gradient magnitude filter

Logarithmic mapping

Exponential intensity remapping

2D/3D local binary pattern (LBP) analysis

3. Wavelet Filtering Protocol

Preprocessed CT images underwent wavelet filtering to transform VOIs into wavelet domain, preserving:

Low-pass (LLL) and high-pass (HHH) subbands

Weighted intermediate subbands (LHL, LHH, LLH, HLL, HHL, HLH)

4. LBP-3D Subcategories

Kurtosis map (LBP-3D-k)

Two spherical harmonic variants: LBP-3D-m1 (Level 1), LBP-3D-m2 (Level 2).

**Supplementary Figure 1** The Kaplan-Meier survival curve for 317 patients with ES-SCLC who were received immunotherapy in the first-line treatment.


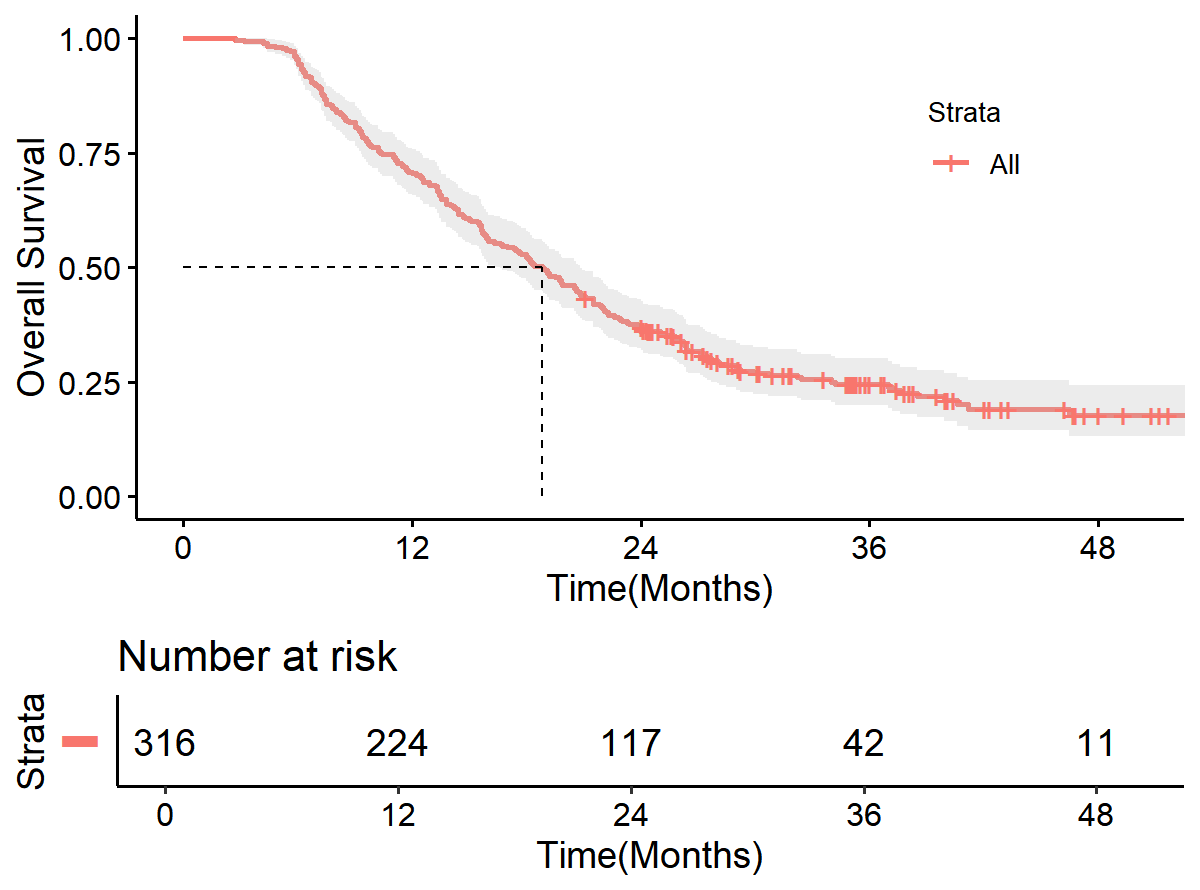


**Supplementary Figure 2** The Kaplan-Meier survival curve demonstrates the difference in survival rates between patients who received TRT and those who didn’t.
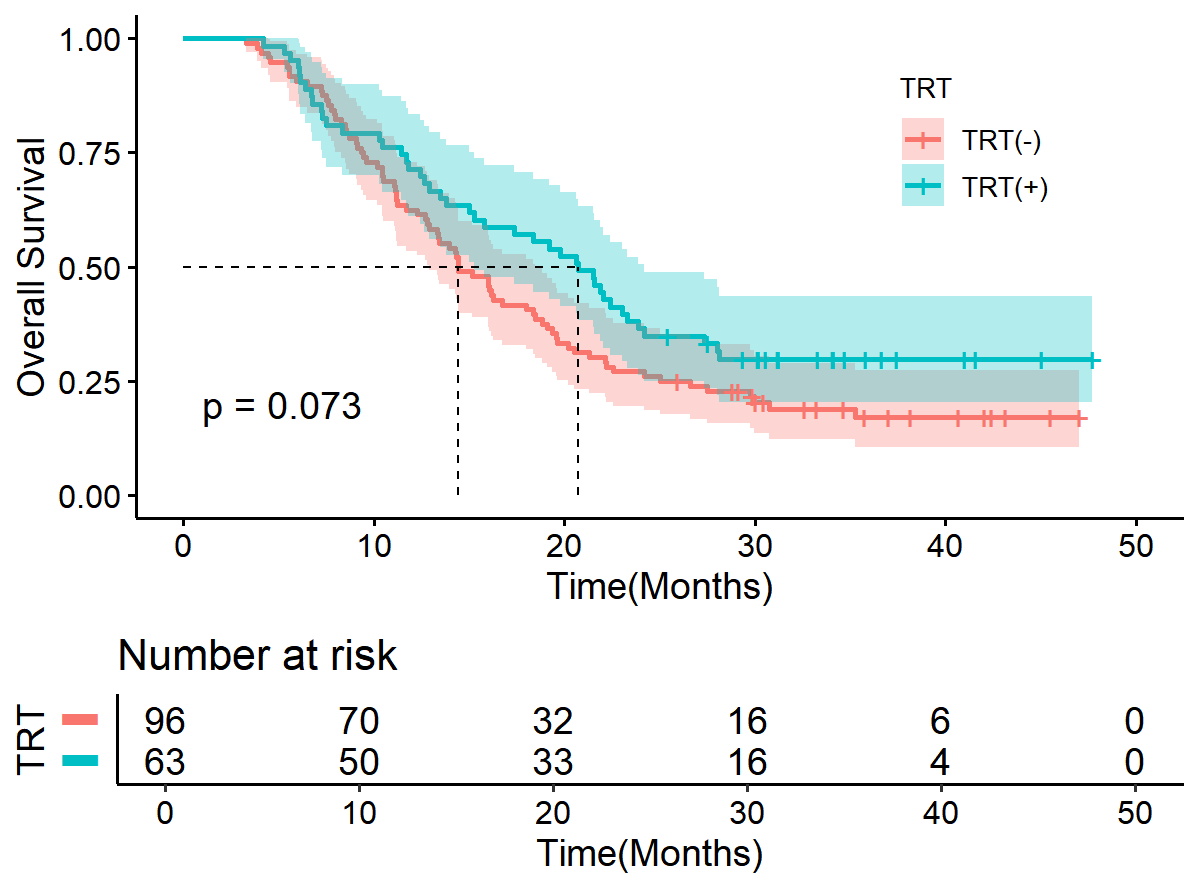


**Supplementary Figure 3** Association Analysis of TLS with OS and combined model.


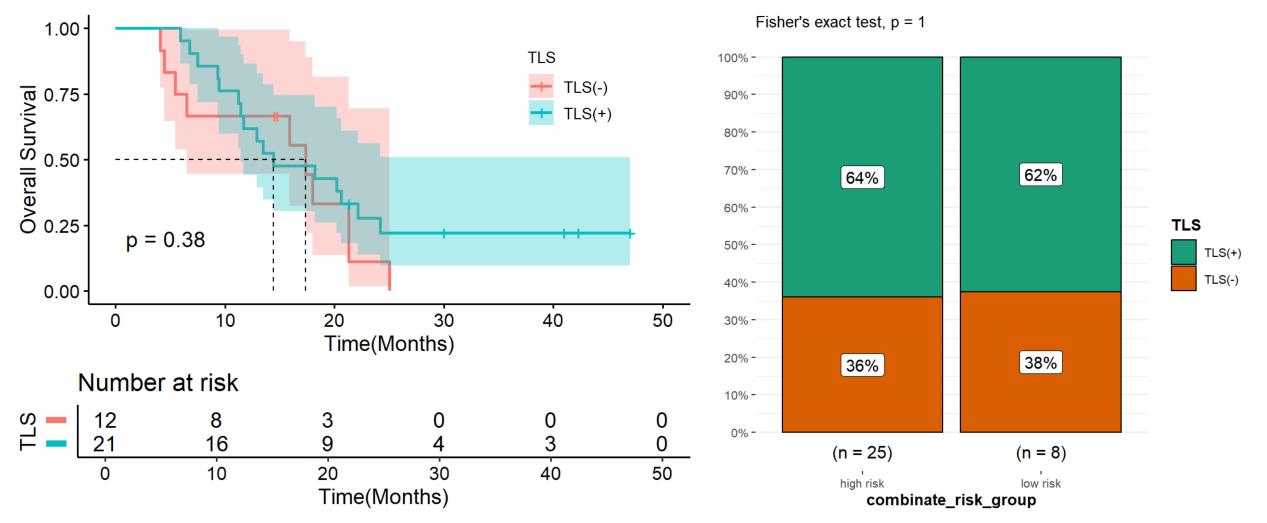


**Supplementary Figure 4** Association analysis of Tumor-infiltrating CD23+ immune cells with OS and combined model.


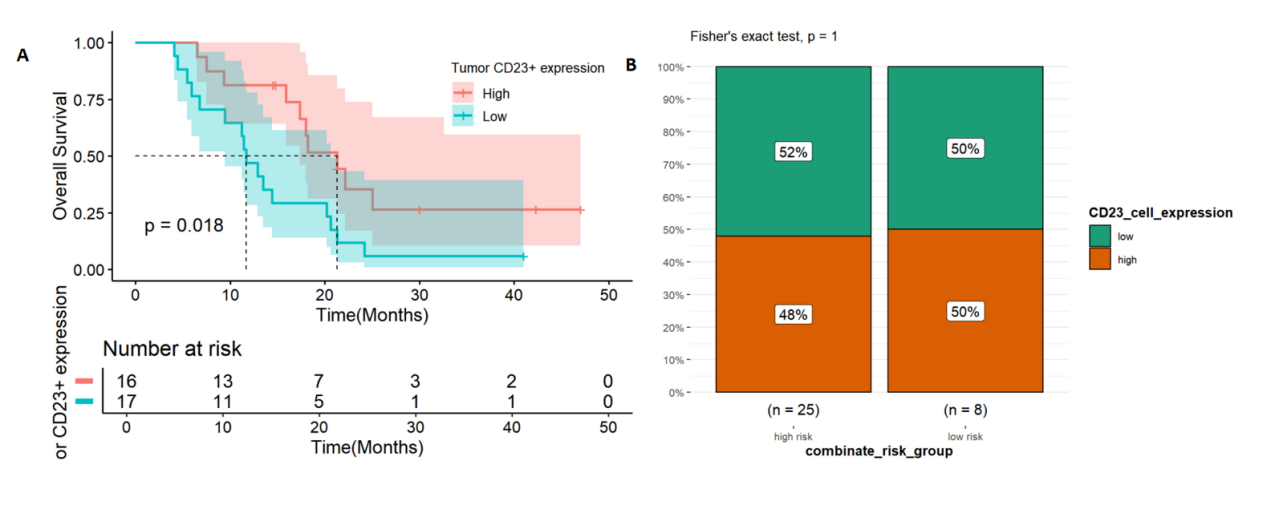


**Supplementary Figure 5** Immune cells within distinct spatial architectures demonstrated potential survival correlations.


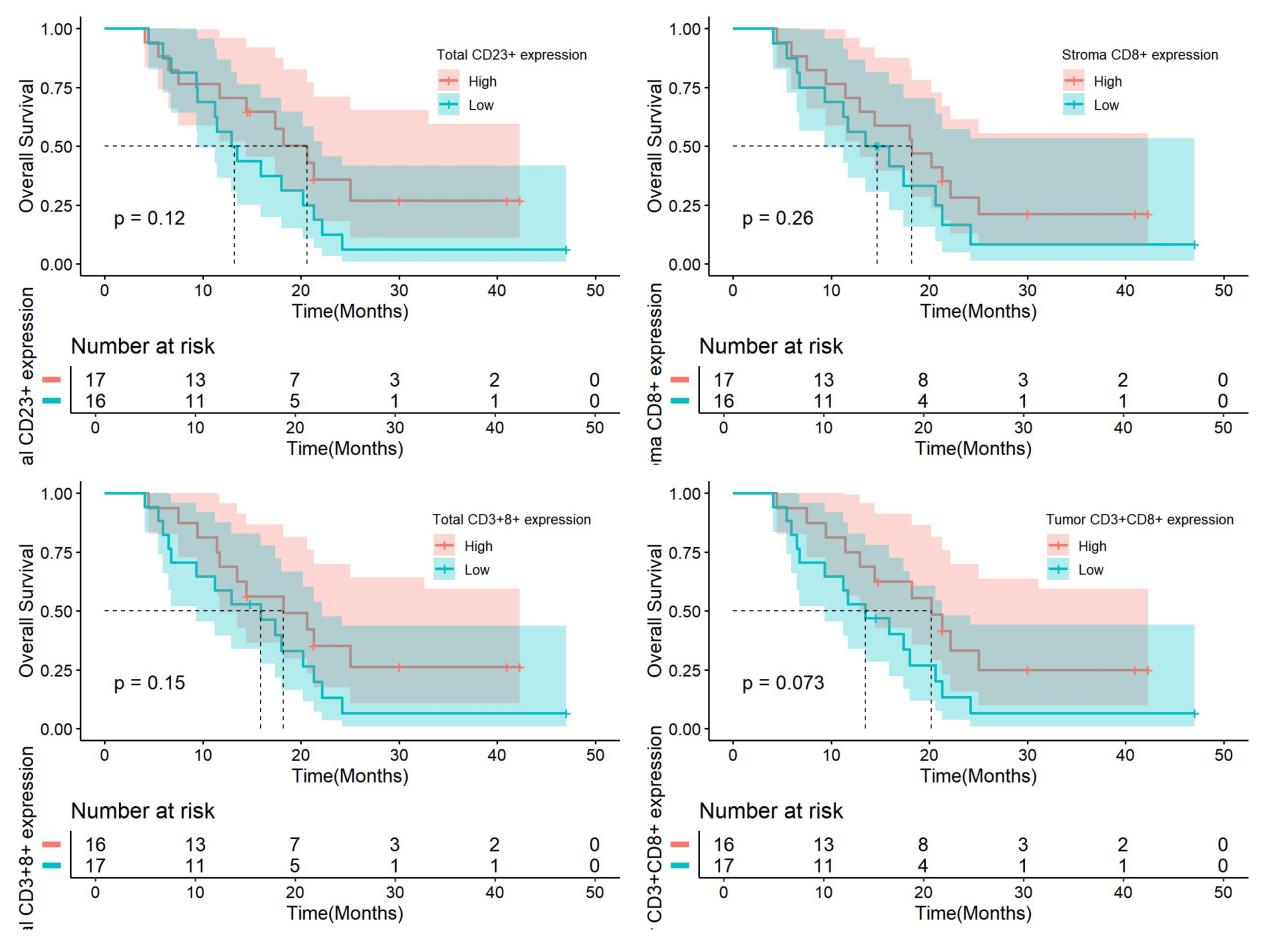


**Supplementary Figure 6** Fisher’s exact test between stromal CD8+ T cells density and the stratified combined risk score groups.


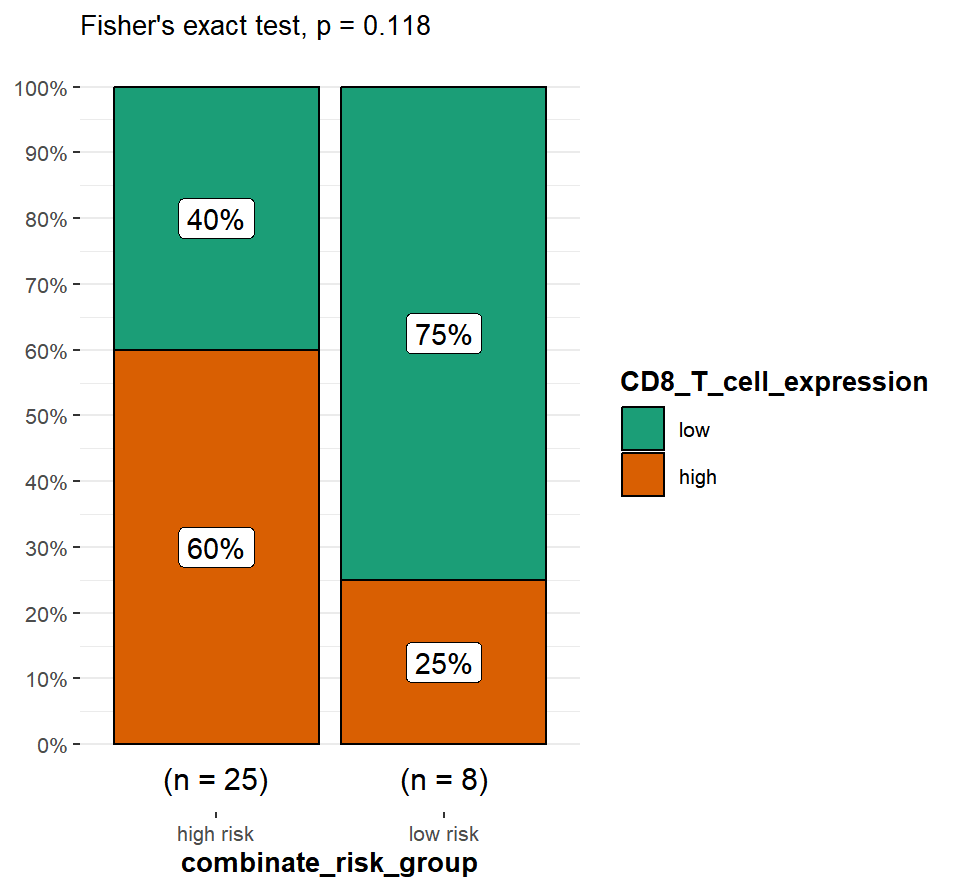


**Supplementary Table 1** Molecular indicators for detection and required antibody information.

| Molecule | Primary antibody product number | Time | Ratio Channel |
| --- | --- | --- | --- |
| CD21 | A8407(abclonal) | 1h | 620 |
| CK | ZM-0069(ZSGB-BIO） | 1h | 480 |
| CD23 | ab92495(Abcam) | 1h | 570 |
| CD20 | ab64088(Abcam) | 1h | 520 |
| CD3 | ab16669(Abcam) | 1h | 690 |
| CD8 | 66868-1-ig(Proteintech) | 1h | 780 |

**Supplementary Table 2** The Wilcoxon rank-sum test to compare the proportions of immune cell types between high combinate score groups and low.

|  | CD3^+^ | CD8^+^ | CD20^+^ | CD21^+^ | CD23^+^ | CD3^+^8^+^ | CD21^+^23^+^ |
| --- | --- | --- | --- | --- | --- | --- | --- |
| Tissue |  |  |  |  |  |  |  |
| P value | 0.190 | 0.352 | 0.374 | 0.758 | 0.696 | 0.091 | 0.726 |
| Stroma |  |  |  |  |  |  |  |
| P value | 0.176 | 0.040 | 0.205 | 0.606 | >0.999 | 0.062 | 0.517 |
| Tumor |  |  |  |  |  |  |  |
| P value | 0.294 | 0.310 | 0.853 | >0.999 | 0.726 | 0.389 | 0.984 |
